# Hyperuricemia and progression of IgA nephropathy: time to consider early uric acid lowering therapy?

**DOI:** 10.1097/MS9.0000000000003783

**Published:** 2025-08-28

**Authors:** Muddassir Khalid, Bushra Gul, Ayesha Nasir, Mohammed Hammad Jaber Amin

**Affiliations:** a Department of Medicine, Nishtar Medical University, Multan, Pakistan; b Department of Medicine, Rawalpindi Medical University, Rawalpindi, Pakistan; c Department of Medicine, Alzaiem Alazhari University, Khartoum, Sudan

*To the Editor,*

IgA nephropathy (IgAN) is the most prevalent primary glomerulonephritis characterized by diffuse mesangial deposition of IgA-or IgA-containing immune complexes and affecting 200 000–350 000 individuals annually worldwide. According to the studies, despite the conventional risk factors including increased proteinuria, renal histopathology and hypertension linked to the progression of IgAN to End Stage Renal Disease ESRD, hyperuricemia and serum uric acid (SUA) have gained attention in recent years as an independent prognostic marker for progressing IgAN**^[1]^.** A retrospective cohort study of 718 biopsy proven IgAN patients shows that every 10 mg/dl increase in the serum uric acid (SUA) level, the risk of renal insufficiency and End-Stage Renal Disease ESRD in IgAN patients is increased by 1.35 times within 5 years during the early stages of the disease**^[2]^.** Pathologically, raised serum uric acid (SUA) levels cause renal injury due to oxidative stress, endothelial dysfunction and activation of renin-angiotensin aldosterone system (RAAS), causing tubular atrophy and interstitial fibrosis, thus worsening the IgAN. Renin-angiotensin-aldosterone system (RAAS) inhibitors have been shown to be beneficial for the long-term renal survival of advanced IgAN, even though blood pressure and proteinuria did not decrease. However, within 20 years of diagnosis, about 30% of IgAN patients over 30 will experience terminal chronic renal failure. Hence, primary prevention strategies like early detection and control of hyperuricemia by urate-lowering therapies (ULTs) is required to protect against the poor renal outcomes of progressing IgAN and its consequences**^[3]^.**

Despite the fact that hyperuricemia is known to be an independent risk factor for renal progression, the clinical utility of ULTs in IgAN is unknown due to mixed results. Various animal studies discovered that xanthine oxidase XO inhibitors like febuxostat suppress the IgAN due to its anti-inflammatory and anti-fibrotic effects**^[4]^.** The FEATHER study, a randomized control trial of XO inhibitor febuxostat in stage 3 CKD patients with asymptomatic hyperuricemia, demonstrated a trend towards control of blood pressure, reduced risks of kidney events and a slower decline in eGFR and decrease the urine albumin to creatinine ratio. Although statistical significance was not achieved in the initial outcomes, yet mitigated decline of kidney function**^[5]^.** Given the existing observational evidence, early ULT may be a reasonable adjunctive strategy for hyperuricemic IgAN patients because of their effectiveness in delaying the progression of kidney function deterioration. However, we advocate targeted RCTs focusing on renal survival, proteinuria and blood pressure control are needed to gain a better understanding of efficacy of hyperuricemia lowering therapy in IgAN patients. Till then, the physicians should remain alert for elevated SUA levels in IgAN patients and consider individualized risk based approaches.

TITAN Guidelines: This manuscript is in compliant to TITAN Guidelines, 2025, declaring no use of AI**^[6]^.**

## Data Availability

None.

## References

[1] Zhang K Tang L Jiang S. Is hyperuricemia an independent prognostic factor for IgA nephropathy: a systematic review and meta-analysis of observational cohort studies. Ren Fail 2022;44:70–80. 35156903 10.1080/0886022X.2021.2019589 PMC8856039

[2] Qi C Liu X Mao J The time-averaged serum uric acid can better predict the prognosis of IgA nephropathy. Nutrition Metabolism and Cardiovascular Diseases [Internet]. 2024 Nov 1 [cited 2025 Jul 27];103800. Available from: https://www.nmcd-journal.com/article/S0939-4753(24)00434-4/fulltext 10.1016/j.numecd.2024.103800 39674719

[3] Liu B Zhao L Yang Q. Hyperuricemia and hypertriglyceridemia indicate tubular atrophy/interstitial fibrosis in patients with IgA nephropathy and membranous nephropathy. Int Urol Nephrol 2021;53:2321–32. 33895976 10.1007/s11255-021-02844-4

[4] Inoue MK Yamamotoya T Nakatsu Y. The xanthine oxidase inhibitor febuxostat suppresses the progression of IgA nephropathy, possibly via its anti-inflammatory and anti-fibrotic effects in the gddY mouse model. Int J Mol Sci 2018;19:3967. 30544662 10.3390/ijms19123967 PMC6320819

[5] Kataoka H Mochizuki T Ohara M. Urate-lowering therapy for CKD patients with asymptomatic hyperuricemia without proteinuria elucidated by attribute-based research in the FEATHER Study. Sci Rep 2022;12:3784. 35260678 10.1038/s41598-022-07737-9 PMC8904814

[6] Agha R Mathew G Rashid R. Transparency in the reporting of artificial intelligence – the TITAN guideline. Premi J Sci 2025;10:100082.

